# A Real Time QRS Detection Algorithm Based on ET and PD Controlled Threshold Strategy

**DOI:** 10.3390/s20144003

**Published:** 2020-07-18

**Authors:** Aiyun Chen, Yidan Zhang, Mengxin Zhang, Wenhan Liu, Sheng Chang, Hao Wang, Jin He, Qijun Huang

**Affiliations:** School of Physics and Technology, Wuhan University, Wuhan 430072, China; aiyunch@whu.edu.cn (A.C.); yidan_zhang@whu.edu.cn (Y.Z.); zhangmengxin@whu.edu.cn (M.Z.); whliu@whu.edu.cn (W.L.); changsheng@whu.edu.cn (S.C.); wanghao@whu.edu.cn (H.W.); jin.he@whu.edu.cn (J.H.)

**Keywords:** electrocardiogram (ECG), exponential transform (ET), PD-control, QRS detection, real-time

## Abstract

As one of the important components of electrocardiogram (ECG) signals, QRS signal represents the basic characteristics of ECG signals. The detection of QRS waves is also an essential step for ECG signal analysis. In order to further meet the clinical needs for the accuracy and real-time detection of QRS waves, a simple, fast, reliable, and hardware-friendly algorithm for real-time QRS detection is proposed. The exponential transform (ET) and proportional-derivative (PD) control-based adaptive threshold are designed to detect QRS-complex. The proposed ET can effectively narrow the magnitude difference of QRS peaks, and the PD control-based method can adaptively adjust the current threshold for QRS detection according to thresholds of previous two windows and predefined minimal threshold. The ECG signals from MIT-BIH databases are used to evaluate the performance of the proposed algorithm. The overall sensitivity, positive predictivity, and accuracy for QRS detection are 99.90%, 99.92%, and 99.82%, respectively. It is also implemented on Altera Cyclone V 5CSEMA5F31C6 Field Programmable Gate Array (FPGA). The time consumed for a 30-min ECG record is approximately 1.3 s. It indicates that the proposed algorithm can be used for wearable heart rate monitoring and automatic ECG analysis.

## 1. Introduction

Electrocardiogram (ECG) plays an important role in the diagnosis of cardiac diseases. To analyze massive ECG data from long-time monitoring system for cardiac condition, such as 24 h Holter (or even longer), it is a heavy and tedious burden for the physicians. Moreover, the occurrence of heart disease is unexpected. To alarm ECG abnormalities in time, the real-time monitoring of the heart condition and automatic analysis of ECG signal is of great significance. The performance of an automatic ECG analysis system relies much on the features extracted from fiducial points such as P, Q, R, S, T, of which the QRS-complex stands out for its large amplitude and sharp slope. Thus, QRS-complex detection has served as the fundamental step for the automatic detection of other ECG fiducial points and further analysis. The morphology of ECG varies greatly from person to person, even in different time for the same individual. In addition, ECG is generally contaminated with various noises, such as power line interference, electrode contact noise, motion artifact, muscle contraction, and baseline wander. These factors have added challenges for automatic QRS-complex detection.

Many efforts have been made on the automatic detection of QRS-complex in decades. Numerous QRS-complex detection algorithms have been proposed in the literature, such as first-order derivative [1,2,3,4], wavelet transform [5,6,7,8,9,10], mathematical morphology [9,10,11], Hilbert transform [12,13,14,15,16], neural network [17,18], etc. Based on the large amplitude and sharp slope features of QRS-complex, Pan & Tompkins [1] realized automatic QRS-complex detection through first-order derivative, non-linear transform, and amplitude/noise threshold, but the accuracy is not so desirable. Using time domain features, Yeh & Wang [3] also proposed the difference operation method (DOM) algorithm. Complex preprocessing techniques is applied in the DOM algorithm, such as the morphological filter, which are time-consuming. Li et al. [5] first introduced wavelet transform to automatic QRS-complex detection, which decomposes ECG signal into components of different time and frequency scales. Then combining both frequency and time domain information and calculating singular degree, it achieved high accuracy for QRS-complex detection, but high complexity as well. Based on [5], Martínez et al. [6] removed singularity analysis and taking any possible QRS morphology into consideration, it searched both positive-negative and negative-positive zero-crossing points. It lowered the complexity of algorithm proposed in [5]. But, wavelet transform methods are time-consuming and have the choice problem of mother wavelet and scales to obtain QRS events [14]. M. Sabarimalai et al. [14] introduced a novel R-peak detector utilizing Shannon energy estimation and Hilbert transform. The algorithm shows great performance in terms of accuracy and speed. However, to implement the Hilbert transform for real-time ECG monitoring, a very long memory buffer is needed to calculate Hilbert transform by sampling all the ECG data and it induces a long delay as well. Moreover, the detection of small and wide QRS-complexes still remains as a problem [19]. Thus, a comprehensive algorithm with good performance both on accuracy and complexity, applicable for hardware implementation is still in need for the real-time detection of QRS-complex.

This paper presents a simple, fast, hardware-friendly, real-time QRS detection algorithm that can be lightweight. The algorithm is based on exponential transformation (ET) and proportional differential (PD) control adaptive threshold strategy, which can effectively detect small and wide QRS-complexes, thereby significantly improving the accuracy and robustness of QRS-complex detection. The rest of the paper is structured as follows. Section 2 describes the proposed algorithm based on ET and PD-controlled adaptive threshold method in detail. The performance of the proposed algorithm testing on MIT-BIH Arrhythmia Database is given in Section 3, followed by discussion in Section 4. Finally conclusion is drawn in Section 5.

## 2. Methods

**Figure 1:**
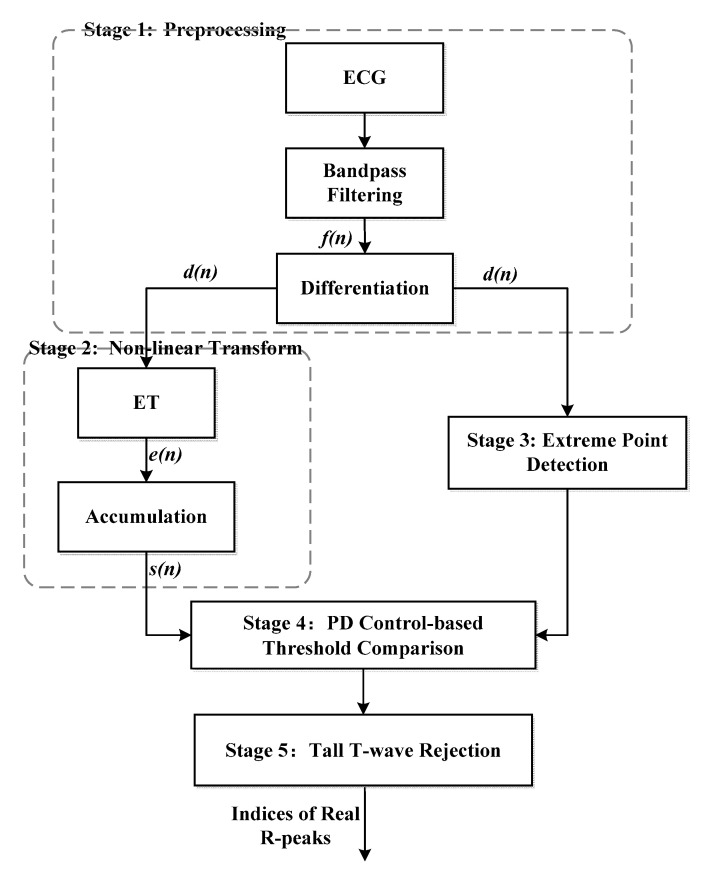
Block diagram of the proposed 5-stage algorithm for QRS-complex detection.

The proposed algorithm consists of five stages, namely, preprocessing, non-linear transform, extreme point detection, PD control-based threshold comparison, and tall T-wave rejection. The preprocessing stage includes a band-pass filter and first-order forward difference to enhance the QRS-complex. The non-linear transform stage first proposes the exponential transform (ET) to narrow the gap between high and low amplitude QRS-complexes, which plays an important role in the detection of small and wide QRS-complex. The third stage uses the sharp slope and high amplitude features of the QRS-complex to select the extreme points as QRS candidates. The fourth stage utilizes PD control-based adaptive threshold to further screen the QRS candidates. The mistaken tall T-waves as QRS-complexes are rejected according to the average RR interval in the last stage. The block diagram of the proposed algorithm is shown in Figure 1.

### 2.1. Preprocessing

To enhance QRS-complex and reduce high frequency muscle contraction noise and low frequency baseline wander, a finite impulse response (FIR) band-pass filter using Hamming window is introduced. Due to the frequency spectrum of QRS-complex, the high cut-off frequency and low cut-off frequency of the filter is 15 and 5 Hz, respectively. According to the characteristics of the signal in this work and after multiple parameter adjustments, the order of the filter is set 41. The group delay of the 41th Hamming window filter is (41−1)/2=20 sampling points. After filtering, on the one hand, the filtered signal f(n) is used to look for extreme points through a sliding window, which will be presented in detail in Section 2.3. On the other hand, the filtered signal is differentiated to catch the steep spikes of QRS-complex. The differentiation of the filtered ECG is implemented as,

(1) d(n)=f(n+1)−f(n)

where f(n) and d(n) represent the filtered signal value and differentiated signal value at point n, respectively. This operation acts as a high-pass filter, which can reduce the interference of tall P and T waves.

### 2.2. Nonlinear Transform

After differentiation, considering the polarity of the QRS-complexes, a non-linear exponential transform (ET) is used to convert both positive and negative peaks into positive peaks. The equation for the ET is as per Equation (2). To facilitate hardware implementation, the exponential transform can be approximated with first-order Taylor expansion in the proposed algorithm.

(2) $$e(n)=\left|d(n)\right|e^{-|d(n)|}\;\approx \left|d(n)\right|-d{\left(n\right)}^{2}$$

**Figure 2:**
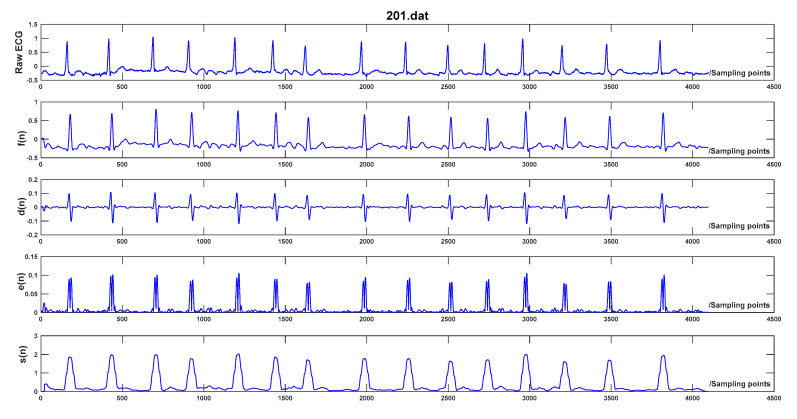
Preprocessing of record 201 in MIT-BIH Arrhythmia Database, f(n) represents the filtered ECG, d(n) stands for the differential ECG, e(n) is the signal after exponential transformation, and s(n) is the accumulation result.

Finally, the transformed signal’s accumulation s[n] is calculated to enhance and smooth e[n] of QRS-complex as follows,

(3) $$s(n)=\sum_{i=n-\left\lfloor \frac{q}{2}\right\rfloor }^{n+\left\lfloor \frac{q}{2}\right\rfloor }e(i)$$

where q represents the product of normal QRS-complex duration (120 ms) and sampling frequency, and $\left\lfloor \frac{q}{2}\right\rfloor$ corresponds to the largest integer less than or equal to q/2. The processing result of each step is illustrated in Figure 2.

**Figure 3:**
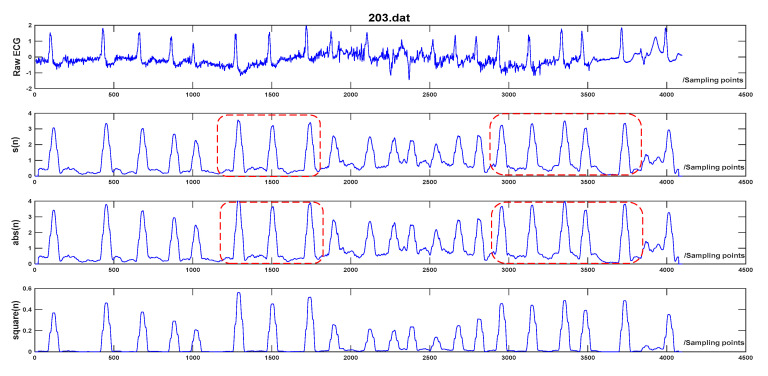
Comparison of three non-linear transforms. The first one is raw ECG of record 203 in the MIT-BIH Arrhythmia Database. The second one to the fourth are the results of using exponential transform, absolute value, and squarer value, respectively. It can be noticed that the amplitude of the signal after ET is compressed compared to the absolute value, especially in the red box. The amplitude deviation after ET is the smallest among the three transforms.

The proposal of ET can effectively narrow the magnitude difference of QRS peaks compared to other non-linear transforms such as squarer value [1,2] and absolute value [4,11], which are widely used in other QRS detection algorithms. The equations for absolute value and squarer value are expressed as Equations (4) and (5). The advantage of the proposed ET is as following: the negative exponential operation $e^{-\left|d\left(n\right)\right|}$ works as an amplitude compression ratio on the differential signal, and the amplitude compression ratio is in proportion to d(n). When d(n) is larger, the amplitude compression ratio gets lager. Thus, the amplitude deviation between the successive peaks gets diminished after ET. It is beneficial to the follow-up PD control-based adaptive threshold strategy and the detection of low amplitude and wide QRS-complex, the reasons will be given in Section 2.4. Instead, the amplitude deviation between the successive peaks increases through square operation and remains the same through absolute operation. The result of the three transforms on Record 203 in MIT-BIH Arrhythmia Database is demonstrated in Figure 3. It presents that the amplitude of R peaks using the proposed transform is more balanced than the other two transforms and the amplitude deviation is the smallest among the three transforms.

(4) $$\mathrm{square}(n)=\sum_{i=n-\left\lfloor \frac{q}{2}\right\rfloor }^{n+\left\lfloor \frac{q}{2}\right\rfloor }|d(i){|}^{2}$$

(5) $$\mathrm{abs}(n)=\sum_{i=n-\left\lfloor \frac{q}{2}\right\rfloor }^{n+\left\lfloor \frac{q}{2}\right\rfloor }\left|d(i)\right|$$

### 2.3. Extreme Point Detection

To detect the QRS-complex, the extreme points which could be possible QRS peaks are first detected. Extreme points are searched within a length-fixed sliding window in the filtered signal f(n). It is known that the normal RR interval (time span between two successive R peaks) is 0.4 s~1.2 s. It is rarely shorter than 240 ms, or the heart rate would be above 250 beats/min. That is to say, in a searching window around 240 ms, there would be one QRS-complex at most. As a consequence, the length of the sliding window is approximately 240 ms. Setting the length of the sliding window from 220 ms to 280 ms, the performance of the proposed algorithm is evaluated and the optimal length is fixed as 260 ms by trial and error.

The point n in the window following the criteria below will be picked out as an extreme point,

(6) $$(f(n)-f(n+j))\times (f(n)-f(n-j))>0,\;j=1,2,\ldots ,\left\lfloor \frac{q}{2}\right\rfloor$$

where f(n) is the amplitude of point n in the filtered signal. Considering that both two ends of the window might also be extreme points as well, the $\left\lfloor \frac{q}{2}\right\rfloor$ points before and after the current window are also included in the window.

### 2.4. Proportional-Derivative (PD) Control-Based Threshold Adjustment

The PD control-based threshold adjustment in [4] is modified to detect the possible QRS peaks. The threshold can be adjusted according to thresholds in the previous two windows and predefined minimum threshold. The threshold at the first point in sliding window w to look for QRS peaks adjusts following the equation below,

(7) $$\mathrm{TH}[w]=\mathrm{TH}[w-1]-a\times (\mathrm{TH}[w-1]-TH_{\mathrm{mih}})\;-b\times (\mathrm{TH}[w-1]-\mathrm{TH}[w-2])$$

where a and b are constants. TH[w], TH[w − 1], and TH[w − 2] are the threshold at the first point in the wth window, (w − 1) th and (w − 2) th window, respectively. THmin is the predefined minimal threshold to prevent mistaking noise as possible a QRS peak.

After calculation of TH[w] and TH[w − 1], the threshold corresponding to each point in window w−1 can be deduced through linear interpolation. Considering the delay of differential and non-linear transform in preprocessing, once there exists one point within 15 points around the extreme point of which the corresponding value s[n] is M times greater than the threshold at the extreme point, the corresponding extreme point is selected as a QRS candidate. Here, M denotes a constant. If there are more than one possible QRS candidates in the same window or the time interval between two successive QRS candidates is within 260 ms, the point with the largest exponential transform and combination value s[n] is selected as the QRS candidate, and the others will be left out. Once a QRS candidate is detected in window w, the first threshold in window w, (i.e., TH[w]) is updated as the exponential transform and accumulation value s[n] of the detected QRS candidate. Because thresholds decrease constantly in the searching window till reaching the minimal threshold in the PD control-based threshold adjustment, a smaller amplitude deviation among the QRS-complex is beneficial to the detection of low amplitude and wide QRS-complex. Thus, the combination of ET and PD control-based threshold adjustment can effectively avoid noise peaks and improve detection results towards small and wide QRS-complex. After many experiments, the empirical value for THmin, a, b, and M are 0.15, 0.5, 0.1, and 1.5, respectively. The pseudo code of the PD control-based threshold adjustment in window w is presented in Table 1.

### 2.5. Tall T Wave Rejection

Since the length of the sliding window is 260 ms, the QRS-complex and tall T wave in the same beat may be both recognized as QRS candidates in some cases. To solve the problem, another threshold meanRR/K is introduced for tall T wave rejection. Once the RR interval (time span of two successive QRS candidates) is larger than the meanRR/K, the candidate with the larger s(n) is recognized as the QRS-complex while the smaller one is rejected as the tall T wave. Parameter meanRR is the average value of the detected RR intervals in the analyzed ECG data. K is a constant. Verified by many experiments, the optimal value for K is 3 by trial and error.

## 3. Results

The proposed QRS-complex detection algorithm is evaluated with the widely used MIT-BIH Arrhythmia Database [20], which is a set of standard test materials for the evaluation of QRS detectors and annotated by two cardiologists. The MIT-BIH Arrhythmia Database contains 48 two-channel 30-min ECG records with a sampling rate of 360 Hz. The database comes from the real Holter records and covers signals with various morphologies and noises. It is noted that in record 207, there exists 2-min ventricular flutter segments. Due to its weird morphology, most state-of-the-art QRS detection algorithms [1,2,3,5,6,7,8,9,10,11,12,13,14,15,16,17,18,21,22,23,24,25,26] exclude the whole record of 207 or the 2-min ventricular flutter segments in record 207 in the evaluation using the MIT-BIH Arrhythmia Database.

The proposed algorithm was implemented using MATLAB R2011b on a 3.2-GHz, Intel i5 CPU. Only the first channel of the MIT-BIH Arrhythmia Database is utilized to detect QRS-complex in the evaluation. Three parameters are used to assess the proposed QRS detection algorithm, (i.e., sensitivity (Se), positive predictivity (P+), and accuracy (Acc)).

By comparing with annotations in MIT-BIH Arrhythmia Database, analyzing 2^14 sampling points (around 45 s) each time, Table 2 shows the QRS detection result using the proposed algorithm. The sensitivity, positive predictivity, and accuracy for the whole MIT-BIH Arrhythmia Database is 99.80%, 99.92%, and 99.71%, respectively. Apart from the 2-min ventricular flutter segments in record 207, the Se, P+ and Acc reaches 99.90%, 99.92%, and 99.82%, respectively. In addition, the performance by analyzing all 650,000 sampling points in one time is also evaluated, and the results is given in Table 3. It demonstrates that the obtained Se and P+ is comparative to that by analyzing 2^14 sampling points each time, which manifests the reliability of the proposed algorithm for real-time QRS detection.

**Figure 4:**
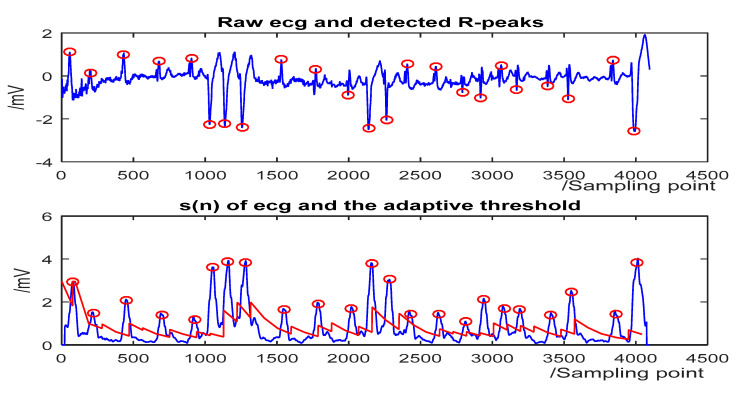
The QRS-complex detection result of record 203 in the MIT-BIH Arrhythmia Database with premature ventricular contractions (PVC) and small normal QRS. (**Top**) the original ECG and detected R-peaks (red circle). (**Bottom**) s(n) in record 203 and the threshold (red line).

**Figure 5:**
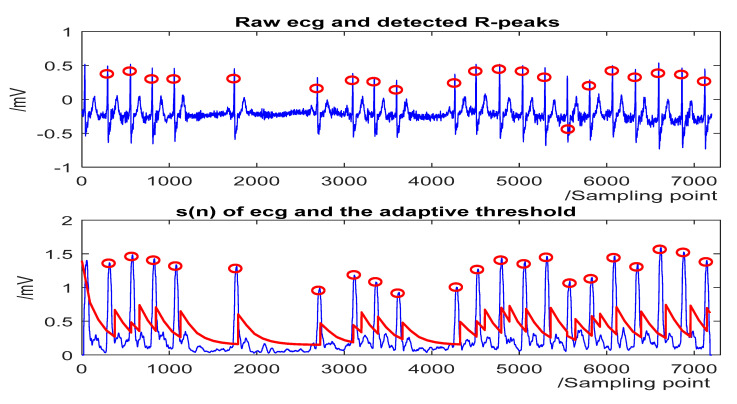
The QRS detection result of record 232 in the MIT-BIH Arrhythmia Database with sudden changes in RR interval and noises. (**Top**) the original ECG and detected R-peaks (red circle). (**Bottom**) s(n) in record 232 and the threshold (red line).

**Figure 6:**
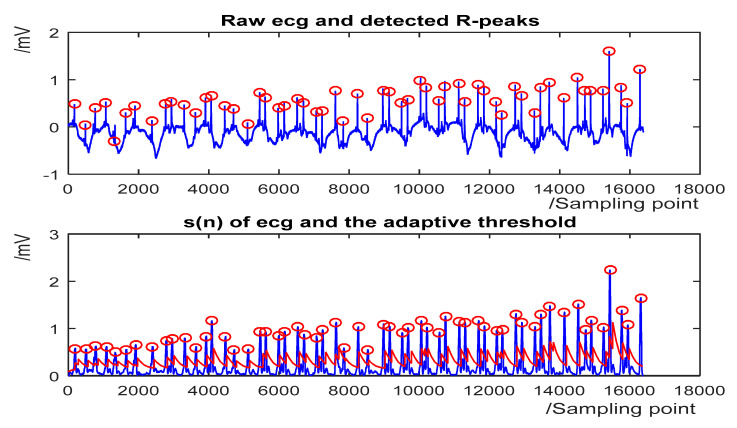
The QRS detection result of record 222 in the MIT-BIH Arrhythmia Database with noise and baseline wander. (**Top**) the original ECG and detected R-peaks (red circle). (**Bottom**) s(n) in record 222 and the threshold (red line).

**Figure 7:**
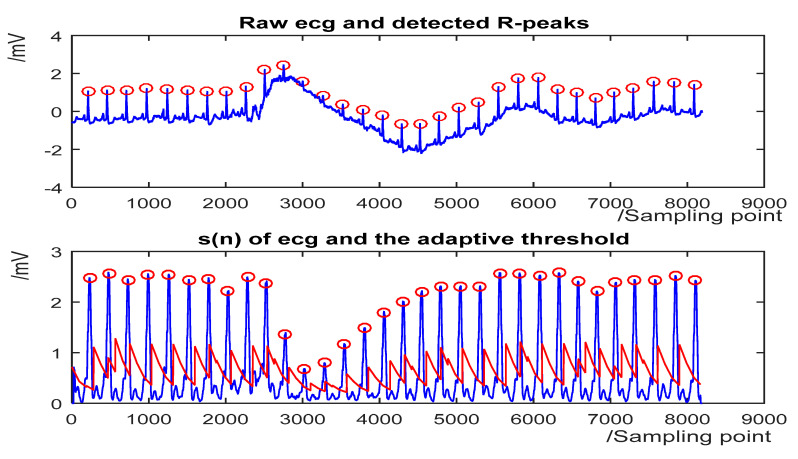
The QRS detection result of record 105 in the MIT-BIH Arrhythmia Database with baseline wander. (**Top**) the original ECG and detected R-peaks (red circle). (**Bottom**) s(n) in record 105 and the threshold (red line).

The detection results for some representative ECG segments with different morphologies and under different SNR are given in Figure 4, Figure 5, Figure 6 and Figure 7. The upper plot is the original ECG and detected R-peaks (red circle), and the lower plot of the s(n) of ECG and the adaptive threshold sequence generated by the PD control-based threshold adjustment is marked with a red line. Figure 4 shows the QRS detection result of record 203 with premature ventricular contractions (PVC) and small normal QRS. As shown in Figure 5, despite the sudden changes in the RR interval and the noise of the signal, most R peaks can be accurately detected. Figure 6 is the result of record 222 with noise and baseline wander. Figure 7 shows the result of Record 105 with baseline wander. It illustrates the good applicability and robustness of the proposed algorithm dealing with different morphologies of ECG and various noise interferences. The average processing time of QRS complex detection for a 30-min ECG record is 1.5 s, and it is fast for real-time QRS detection.

The cause of the relatively high detection error of the proposed algorithm towards some records is also analyzed. Record 105 contains a nearly 1-min ECG segment with extremely poor signal quality. For record 203, there are server QRS morphology changes in the first channel due to axis shifts and considerable noise including muscle artifact and baseline shift. It is a very difficult record, even for cardiologists. Most state-of-the-art QRS-complex detectors also yields relatively low detection accuracy of this records as well. The relatively high detection error of record 207 is due to the 2-min ventricular flutter segments.

**Figure 8:**
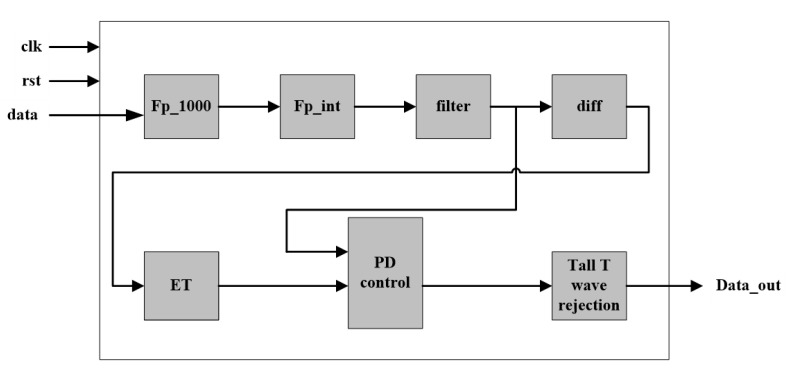
Architecture of the hardware implementation of the proposed algorithm.

The proposed algorithm is also implemented on an Altera Cyclone V 5CSEMA5F31C6 FPGA with Verilog HDL (hardware description language). ECG data in the MIT-BIH Arrhythmia database are stored in an external (synchronous dynamic random-access memory) SDRAM. The architecture of the hardware implementation of the proposed algorithm is given in Figure 8. The data is read at the frequency of 500 KHz and processed at 50 MHz. When the data reading is completes, the data are first magnified 1000 times through floating-point multiplier and converted to integers through the IP core, and then processed by the filter, differential and ET modules. After that, a length-fixed data buffer is created to cache the data after ET for sliding window analysis. Then, the data are processed by PD control-based threshold adjustment and Tall T wave rejection modules, and finally the result output. After completing analysis of the data in the data buffer, the data in the data buffer get updated and go through the next cycle of data reading and analysis. In this process, the implementation of the algorithm utilizes the pipeline design method to read and process the ECG data, so that the ECG data can be analyzed in real time.

Table 4 presents the performance comparison between the hardware implementation and software implementation of the proposed algorithm. The accuracy of the hardware-based algorithm is slightly decreased due to the quantization errors and the accuracy loss get almost negligible. The estimation of the processing time is approximately 1.3 s to achieve the QRS-complex detection of a 30-min ECG record with a 50 MHz system clock. And the processing time for a single beat is about 0.0119 ms. It is validated as well that the performance by is identical to that by using exponential transform. It demonstrates that it is feasible to use first-order Taylor expansion to approximate the exponential transform in the proposed algorithm, which is easier for hardware implementation. The device utilization summary after synthesis is given in Table 5. The performance in terms of speed and resource utilization can be improved by further optimization on hardware architecture and making better use of the characteristics of hardware.

## 4. Discussion

Table 6 lists the R-peak detection performance comparison with other state-of-the-art methods. However, it should be noted that since the tolerance time window to match the automatic and manual annotations varies from each work, the comparisons are not perfectly accurate. For example, in the work of Zalabarria et al. [23], the length of the time window is 150 ms. And in the work of Francisco et al. [24], the length of the time window is 320 ms. To make fair comparison of algorithm performance, a correct detection of the QRS complex is defined as a peak of detected less than 300 ms from the annotation peak in this paper. Table 6 shows that the performance of the proposed algorithm is second only to that of Li et al. [5] in terms of Acc. The computation time comparison with other methods is given in Table 7. Considering the different sampling rate and length of the ECG records, the computation time is normalized by calculating the average processing time for each sampling point.

In our method, the average time to process a 30-min (648,000 sampling points) signal is usually 1.5 s. Therefore, the average time for analyzing each point is 2.31 µs in the proposed algorithm. It demonstrates that the speed of the proposed algorithm outperforms that of all the references. The improvement on processing time would be predominant when dealing with massive ECG data.

Compared to other methods in the literature, the proposed algorithm obtains higher or well-matched detection performance, while it uses simple transform and threshold comparison, which is of less computation load and easy for hardware implementation. First, the arithmetic involved in the algorithm is simple, such as addition, subtraction, multiplication, division, and there is no complex logic and preprocessing in the algorithm. Second, the whole hardware architecture consists of seven modules including ECG data magnification, float-point to integer conversion, filtering, differential, ET, PD-control based threshold adjustment, and tall T wave rejection, which is suitable for the design of the pipeline. Third, the ECG data are magnified 1000 times to be converted from float-point data to integers. Compared to the floating-point operation, the integer operation is of less latency and simpler timing sequence, which simplifies the design and improves the processing efficiency. Moreover, due to the sliding window strategy, a short data buffer is utilized for analyzing each data segment. The processing method of data segmentation not only greatly reduces the resource consumption, but also simplifies the hardware architecture. Searching-back and secondary threshold schemes are also not employed in the proposed algorithm, as well the QRS detection algorithm gets consequently simplified.

## 5. Conclusions

A simple, fast, reliable, and hardware-friendly algorithm for real-time QRS detection is proposed in this paper. The paper first puts forward the exponential transform and combines with the PD control-based threshold strategy, which contributes to a better detection performance towards the QRS-complex, especially for small and wide QRS-complexes. The raw ECG data are processed by a band-pass filter, first-order derivative, exponential transform, and accumulation. A moving window is used to look for extreme points in filtered ECG, and then a PD control-based adaptive threshold is utilized for QRS detection. Testing on the widely recognized MIT-BIH Arrhythmia Database, the proposed algorithm shows prominent comprehensive ability of fast speed and high accuracy. Moreover, this algorithm uses a simple transform and threshold comparison, which is applicable for hardware implementation. It can be concluded that the proposed algorithm in the paper is simple, fast, and hardware-friendly for real-time QRS detection. In the future, the proposed algorithm may have applications in wearable data and automatic ECG analysis.

## Figures and Tables

**Table 1:** The algorithm of the proportional-derivative PD control-based threshold adjustment in window w.

**Input:** position of extreme point positions in window w (ex_position)
**Input:** result of s(n) (s)
**Input:** initial threshold at the first point of window *w − 1* (TH1) **Input:** initial threshold at the first point of window *w* (TH2) **Input:** initial threshold at the first point of window *w + 1* (TH3)
**Output:** flag indicating a possible QRS-complex candidate (F_QRS) **Output:** updated threshold at the first point of window *w* (TH1) **Output:** updated threshold at the first point of window *w + 1* (TH2) **Output:** updated threshold at the first point of window *w + 2* (TH3) M = 1.5; window_length = 0.26 × 360; count = 0; A = 0.5; b = 0.1; TH_min = 0.15; **begin** **for** i = 1 to length(ex_position)
**if** s(ex_position(i)) > M × (TH2 − (TH2 − TH3)/window_length × (ex_position)) **then**
Count = count + 1; **if** count = 1 **then** max_s = s(ex_position(i)); max_pos = ex_position(i); **else if** s(ex_position(i)) > max_s **then** max_s = s(ex_postion(i)); max_pos = ex_position(i); **end** **end** **end** **end** **if** count >= 1 **then** F_QRS = true; TH2 = max_s; TH3 = TH2 − a × (TH2 − TH_min) − b × (TH2 − TH1); **end** temp1 = TH3; temp2 = TH2; TH3 = TH3 − a × (TH3 − TH_min) − b × (TH3 − TH1); TH2 = temp1; TH1 = temp2; **return** F_QRS; **return** TH3; **return** TH2; **return** TH1; **end**

**Table 2:** Results of evaluating the proposed QRS-complex detection algorithm using MIT-BIH arrhythmia database.

Tape (No.)	Total (Beats)	TP	FP	FN	Se (%)	P+ (%)	Acc (%)
100	2273	2273	0	0	100.00	100.00	100.00
101	1865	1865	1	0	100.00	99.95	99.95
102	2187	2187	0	0	100.00	100.00	100.00
103	2084	2084	0	0	100.00	100.00	100.00
104	2230	2230	6	0	100.00	99.73	99.73
105	2572	2572	32	0	100.00	98.77	98.77
106	2027	2027	2	0	100.00	99.90	99.90
107	2137	2136	0	1	99.95	100.00	99.95
108	1763	1763	4	0	100.00	99.77	99.77
109	2532	2532	0	0	100.00	100.00	100.00
111	2124	2123	0	1	99.95	100.00	99.95
112	2539	2539	0	0	100.00	100.00	100.00
113	1795	1795	0	0	100.00	100.00	100.00
114	1879	1878	3	1	99.95	99.84	99.79
115	1953	1953	0	0	100.00	100.00	100.00
116	2412	2397	1	15	99.38	99.96	99.34
117	1535	1535	0	0	100.00	100.00	100.00
118	2278	2278	1	0	100.00	99.96	99.96
119	1987	1987	0	0	100.00	100.00	100.00
121	1863	1862	0	1	99.95	100.00	99.95
122	2476	2476	0	0	100.00	100.00	100.00
123	1518	1518	0	0	100.00	100.00	100.00
124	1618	1618	0	0	100.00	100.00	100.00
200	2601	2598	0	3	99.88	100.00	99.88
201	1963	1943	1	20	98.98	99.95	98.93
202	2136	2134	0	2	99.91	100.00	99.91
203	2980	2957	6	23	99.23	99.80	99.03
205	2656	2217	0	2	99.91	100.00	99.91
207	2332	2217	2	115	95.07	99.91	94.99
208	2955	2946	3	9	99.70	99.90	99.59
209	3005	3005	1	0	100.00	99.97	99.97
210	2650	2632	4	18	99.32	99.85	99.17
212	2748	2748	0	0	100.00	100.00	100.00
213	3251	3250	0	1	99.97	100.00	99.97
214	2262	2262	1	0	100.00	99.96	99.96
215	3363	3363	0	0	100.00	100.00	100.00
217	2208	2208	2	0	100.00	99.91	99.91
219	2154	2153	0	1	99.95	100.00	99.95
220	2048	2048	0	0	100.00	100.00	100.00
221	2427	2427	0	0	100.00	100.00	100.00
222	2483	2474	2	9	99.64	99.92	99.56
223	2605	2604	0	1	99.96	100.00	99.96
228	2053	2053	13	0	100.00	99.37	99.37
230	2256	2256	0	0	100.00	100.00	100.00
231	1571	1571	0	0	100.00	100.00	100.00
232	1780	1780	7	0	100.00	99.61	99.61
233	3079	3078	0	1	99.97	100.00	99.97
234	2753	2753	0	0	100.00	100.00	100.00
Total	109,966	109,742	91	224	99.80	99.92	99.71

**Table 3:** R-peak detection performance comparison by different analyzing units.

Analyzing Unit (Sampling Points)	Total (Beats)	TP (Beats)	FN (Beats)	FP (Beats)	Se (%)	P+ (%)	Acc (%)
2^14	109,496 *	109,386	110	91	99.90	99.92	99.82
650,000	109,496 *	109,380	116	93	99.89	99.92	99.81

* The total heartbeats in the MIT-BIH Arrhythmia Database excluding 2-min ventricular flutter segments in record 207.

**Table 4:** R-peak detection performance comparison. Between software implementation and hardware implementation.

Implementation	Analyzing Unit (Sampling Points)	Total (Beats)	TP (Beats)	FN (Beats)	FP (Beats)	Se (%)	P+ (%)	Acc (%)	Time for Processing a Single Record (s)	Time for Processing a Single Beat (ms)
Software	650,000	109,496 *	109,380	116	93	99.89	99.92	99.81	1.5	0.0137
Hardware	650,000	109,496 *	109,326	170	73	99.85	99.93	99.78	1.3	0.0119

* the total heartbeats in the MIT-BIH Arrhythmia Database excluding 2-min ventricular flutter segments in record 207.

**Table 5:** R-peak detection performance comparison between software implementation and hardware implementation.

Source	Amount	Occupancy Rate
Logic utilization (in ALMs)	22,332/32,070	70%
Memory bit	262,144/4,065,280	6%
DSP blocks	87/87	100%

**Table 6:** R-peak detection performance comparison with other methods.

QRS Detector	Total (Beats)	TP (Beats)	FN (Beats)	FP (Beats)	Se (%)	P+ (%)	Acc (%)
Pan & Tompkins [1]	116,137	106,025	277	507	99.75	99.54	99.33
Kim & Shin [22]	109,494	109,357	107	97	99.90	99.91	99.81
Zalabarria et al. [23]	106,581	106,096	485	431	99.54	99.60	99.14
El Bouny et al. [25]	109,494	109,316	178	147	99.84	99.87	99.70
M.Sabarimalai et al. [14]	109,493	109,353	79	140	99.93	99.87	99.79
Sajjad Farashi [27]	109,965	109,692	273	163	99.75	99.85	99.60
Proposed work	109,966	109,736	224	91	99.80	99.92	99.71
Proposed work	109,496 ^b^	109,381	110	91	99.90	99.92	99.82

^b^ the total heartbeats in MIT-BIH Arrhythmia Database excluding 2-min ventricular flutter segments in record 207.

**Table 7:** Computation time comparison with other methods.

QRS Detector	Database	Sampling Rate (Hz)	ECG Data	Time (s)	Time for Analyzing Each Sampling Point (µs)
Li et al. [5]	MIT-BIH Arrhythmia	250	10 min	60	400
Legarreta et al. [2]	MIT-BIH Arrhythmia	360	30 min	25	38.58
Yeh & Wang [3]	MIT-BIH Arrhythmia	360	10 min	22-30	101.85–138.89
Madeiro et al. [21]	QT	250	15 min	4.52	20.09
Poli et al. [26]	MIT-BIH Arrhythmia	360	30 min	No more than 1-2	No more than 1.54–3.08
M.Sabarimalai et al. [14]	MIT-BIH Arrhythmia	360	30 min	2.24	3.45
Proposed work	MIT-BIH Arrhythmia	360	30 min	1.5	2.31
